# Emergence of multi-drug-resistant *Mycobacterium tuberculosis* in Niger: A snapshot based on whole-genome sequencing

**DOI:** 10.1371/journal.pntd.0010443

**Published:** 2022-05-25

**Authors:** Zelika Harouna Hamidou, Madjid Morsli, Saidou Mamadou, Michel Drancourt, Jamal Saad

**Affiliations:** 1 Aix-Marseille-Univ, IRD, MEPHI, IHU Méditerranée Infection, Marseille, France; 2 IHU Méditerranée Infection, Marseille, France; 3 Laboratoire National de Référence des IST/VIH et de la Tuberculose, Niamey, Niger; 4 Université Abdou Moumouni, Niamey, Niger; NIH-National Institute for Research in Tuberculosis-ICER, INDIA

## Abstract

**Background:**

Among other West African countries experiencing the high endemicity of deadly tuberculosis, the situation in Niger is poorly evidenced by microbiological investigations.

**Methodology/Principal findings:**

The study of 42 isolates of *Mycobacterium tuberculosis* from Niger by whole genome sequencing using Illumina iSeq technology yielded four *M*. *tuberculosis* lineages: Indo-Oceanic L1 (n = 1) (2.3%), East-Asian (n = 1) (2.3%), East-African Indian L3 (n = 2) (4.7%) and Euro-American L4 (n = 38) (90.4%). The sub-lineage L4.1.3 comprising 18 isolates (47.3%) was predominant, followed by the L4.6.2.2 sub-lineage (Cameroon genotype, n = 13 isolates) (34.2%). Investigating drug resistance profile for 12 antibiotics found 8/42 (19%) pan-susceptible isolates and 34/42 (81%) resistant isolates; with 40/42 (95.2%) isolates being susceptible to clofazimine-bedaquiline.

**Conclusions/Significance:**

These unprecedented data from Niger highlight the dynamics of tuberculosis transmission and drug resistance in Niger and may assist tuberculosis control in this country which continues to support a high burden of tuberculosis.

## Author summary

Tuberculosis is a major public health problem in Niger, in West Africa. Niger has an emerging problem with multidrug-resistant tuberculosis (MDR-TB). Whole genome sequencing was used to understand the epidemiology of tuberculosis and genetics of multi-drug resistance among patients from the regions in Niger. In this study, most isolates of *M*. *tuberculosis* from this dataset belonged to the L4.6.2.2 sub-lineage and L4.1.3 sub-lineage within the Euro-American lineage. Thirty-four out of 42 (81%) isolates were detected as resistant isolates. Our study highlights the need for epidemiological surveillance and more concerted efforts to ensure that patients are put through treatment.

## Introduction

Niger is a country in West Africa with an estimated 23-million inhabitants. It is experiencing high endemicity of deadly tuberculosis, with 11,485 new and relapsing cases, including 87% of pulmonary forms, which were notified to the World Health Organization in 2019. In Niger, the incidence of multidrug-resistant (MDR)/rifampicin resistance (RR) was estimated at 2.6% (1.2–4.5) per 100,000 inhabitants [1] and in 2019, patients between 25–34 years old were the most affected age group, with a male predominance [1]. As in most limited resource countries, knowledge of the epidemiology of tuberculosis in Niger has been based solely on recording the number of cases as well as baseline demographic characteristics of patients.

The actual incidence of tuberculosis in the various regions of Niger, therefore, is poorly known; and the *Mycobacterium tuberculosis* (*M*. *tuberculosis*) complex (MTBC) species and lineages responsible for laboratory-documented cases, are also poorly known, limiting preventive public health work. The lineage of cases caused by *M*. *tuberculosis* demonstrates distinct geographical associations worldwide [2,3]. The genetic diversity of circulating strains of the *M*. *tuberculosis* complex may have implications for public health, as observed previously in several studies in West African countries [4,5].

Second generation high throughput sequencing technologies (such as the Illumina iSeq 100 System) mean it is now possible to perform whole genome sequencing (WGS) of *M*. *tuberculosis* [6]. Current genomic data showed that MTBC comprises nine human-adapted lineages (lineages 1–9) [4,7–10]. All MTBC lineages have been reported in Africa, suggesting that the MTBC emerged from a common ancestor, and expanded further following human migrations [11–15]. However, to our knowledge, no study has reported any MTBC-WGS of the *M*. *tuberculosis* complex in Niger.

Here, we investigated a collection of MTBC isolates by WGS to gain knowledge of the *M*. *tuberculosis* species, lineages and sub-lineages circulating in Niger, and tentatively correlated them to anonymised demographic data.

## Materials and methods

### Study collection

**Fig 1:**
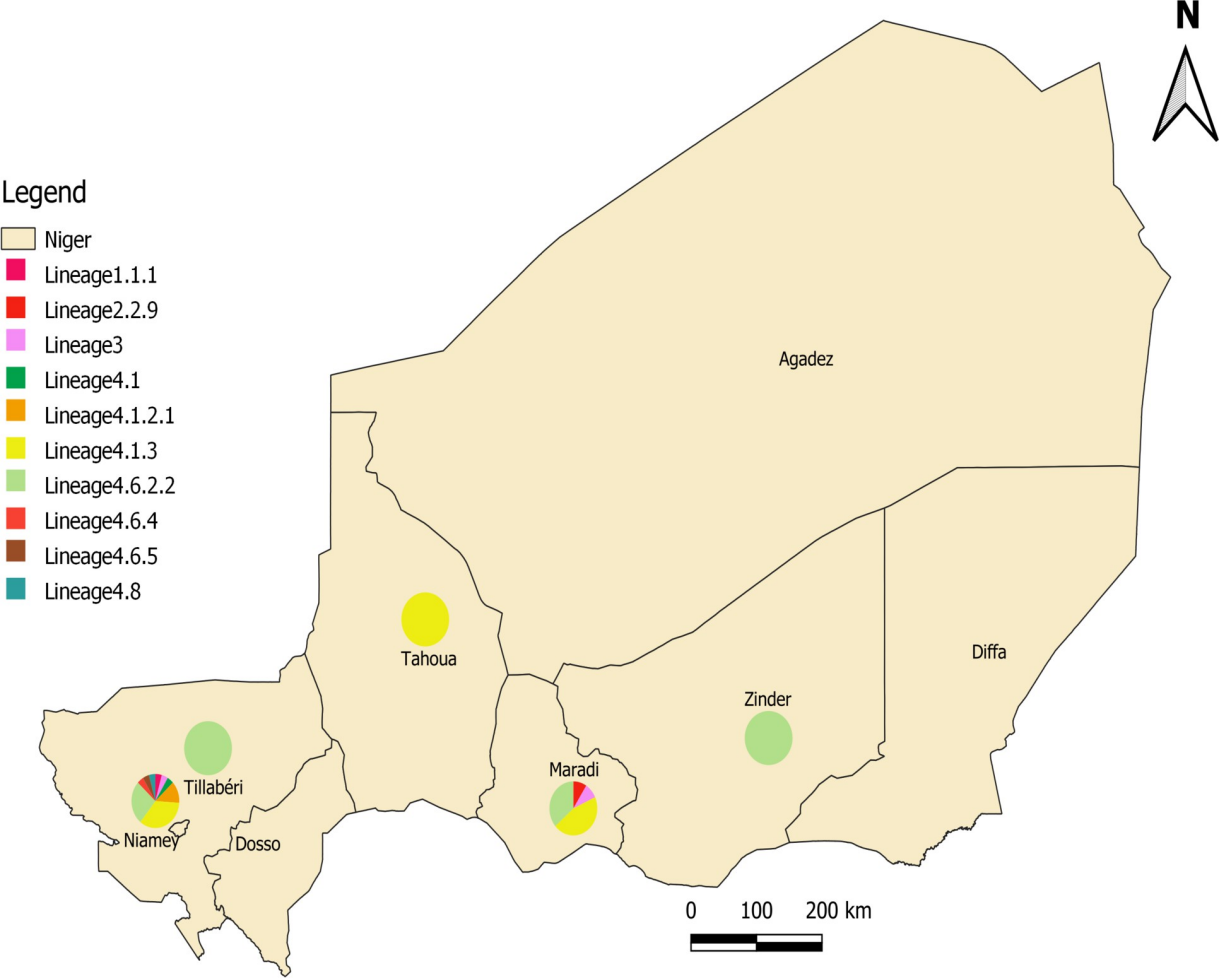
Geographical distribution of *M*. *tuberculosis* lineages/sub-lineages in Niger. Map created with DIVA-GIS (https://www.diva-gis.org/gdata) using GADM version 1.0 data (https://gadm.org/data.html).

This retrospective study included all the positive cultures of mycobacteria routinely made for the diagnosis of pulmonary tuberculosis from respiratory tract specimens collected from patients suspected of having pulmonary tuberculosis, and from monitoring of multidrug resistance patients in Niger in 2016 and 2017. Respiratory tract specimens routinely addressed to the Laboratoire National de Référence des IST/VIH et de la Tuberculose, Niamey, Niger from five regions (Maradi, Niamey, Tahoua, Tillaberi and Zinder) were subjected to smear microscopic examination after auramine-O staining, decontamination using the modified Petroff method [16], and culture on Löwenstein-Jensen medium, prepared according to the manufacturer’s instructions (Merck, Darmstadt, Germany) (Fig 1). Any colonies were verified by Ziehl-Neelsen staining, stored at -20° C and shipped to MEPHI, IHU Méditerranée Infection, Marseille, France for further investigation, as described below. Colonies which did not stain by Ziehl-Neelsen staining were not included in this study. No clinical samples were specifically collected for the present study, samples have been collected as part of the patients’ routine medical management. We obtained permission from the head of the laboratory to carry out any investigation deemed useful on the strains of mycobacteria from our internal library.

### Isolate culture and DNA sequencing

All the laboratory investigations involving non-inactivated isolates were performed in the biosafety level 3 laboratory, at the IHU Méditerranée Infection, Marseille, France. Isolates were subcultured on Middlebrook 7H10 medium incorporating the OADC medium enrichment (oleic acid-albumin-dextrose-catalase) prepared according to the manufacturer’s instructions (Becton Dickinson, Franklin Lakes, USA). Cultures were incubated in an aerobic atmosphere for up to 21 days at 37° C and visually inspected for colonies weekly. A loopful (equivalent to ten 2-microliter inoculation loops) of colony biomass was collected in a 1.5-mL tube containing 200 μL sterile phosphate buffered saline (PBS), and was heat-inactivated at 100°C for one hour [17]. Total DNA was extracted by vortexing the suspension with glass powder (Sigma-Aldrich, St. Louis, MO, USA) using a FastPrep apparatus (MP Biomedicals, Santa Ana California, USA) followed by a Qiagen kit with EZ1 DNA Tissue Kit (Qiagen) according to the manufacturer’s recommendations (Courtaboeuf, France) and eluted in 50-μL volume [18]. The Illumina iSeq library was prepared as previously described [19]. Briefly, DNA (1 ng) was fragmented in a mix containing 5 μL of Amplicon Tagment Mix in the presence of Tagment DNA Buffer (Nextera XT Library prep Kit, Illumina) for five minutes at 55°C, in an ABI 2720 GeneAmp PCR System Thermal Cycler (Applied Biosystems, Foster City, CA, USA) in a 20 μL volume. Then, 5 μL of Neutralize Tagment Buffer was added before centrifugation for one minute at 2,800g and five minutes’ incubation at room temperature, then indexed and amplified in a 50 μL volume, followed by 18 cycles of PCR-index reaction, in the presence of Nextera XT Index Kit V2 (Nextera, San Diego, USA). A first purification was performed using Agencourt Ampure XP beads (Beckman Coulter, Villepinte, France) in a 0.8 ratio of beads followed by two washes with 80% alcohol and elution in 52.5 μL of RSB buffer. The library concentration was measured in Agilent 2100 Bioanalyzer (Thermo Fisher Scientific), then diluted to 100 μL RSB buffer in the presence of 10 μL-volume of Phix (50 pM). Finally, the diluted libraries (50 pM) were denatured and sequenced on the iSeq 100 sequencer (Illumina) in a single 17.5-h run providing 2x150-bp long reads.

Study strains are available in the CSUR collection at IHU laboratory with specific CSUR number of each sample (S1 Table).

### Genome typing and cluster identification

After reads were stored for the delay of analysis, kaiju with default parameters [20] was used to detect for contamination level using NCBI BLAST nr—non-redundant protein database including bacteria, archaea and viruses (2021-02-24 (52 GB). Overall qualities before and after trimming sequencing reads were evaluated using FastQC [21] and multiqc [22]. Then, Trimmomatic tool [23] was used to remove residual Illumina adapters and Illumina-specific sequences. Species, lineages and sub-lineages were identified directly based on output iSeq read using the Tb-profiler (TB_v0.1.3) (https://tbdr.lshtm.ac.uk/) and MTBseq [24] with default settings by specific mapping to reference *M*. *tuberculosis* H37Rv (NC_000962.3). MTBseq was used to recover the statistical mapping data of output sequencing reads using *M*. *tuberculosis* H37Rv (NC_000962.3) as the reference genome (S1 Table). In addition, a local SNPs database constituted as previously reported, was used to identify Beijing sub-lineages [12,25,26]. Genome sequences were assembled using SPAdes version 3.13.1 [27] and annotated using Prokka version 1.12 [28]. The Roary pangenome pipeline [29] was used to generate the core-genes alignment of 42 clinical strains to create the phylogenetic tree in order to represented study strains using following parameters: 80% minimum identity for blastp, and a gene detected in 99% of isolates to be recorded as a core gene. Phylogenetic trees based on core genome were generated using FastTree 2.1.10_1 software (https://ngphylogeny.fr/tools/tool/271/form) using GTR model and bootstrap option with 1000 replicates.

### Detection of drug resistance

The Tb-profiler was used to recover *in silico* susceptibility-resistance profiles to isoniazid, rifampicin, ethambutol, pyrazinamide, ethionamide, fluoroquinolones, streptomycin, capreomycin, amikacin and kanamycin. Each detected mutation was confirmed by mapping the output sequencing reads to the reference *M*. *tuberculosis* H37Rv resistance genes. Resistance mutations of *atp*C, *atp*E, rv0678, rv1979c and rv2535c were susceptibility searched [30–36] by mapping the output sequencing reads against these genes to detect resistance to clofazimine and bedaquiline (S2 Table). The impact of mutations on protein function was estimated using the PROVEAN score (http://provean.jcvi.org/seq_submit.php).

## Results

### *M*. *tuberculosis* genomic analysis

**Fig 2:**
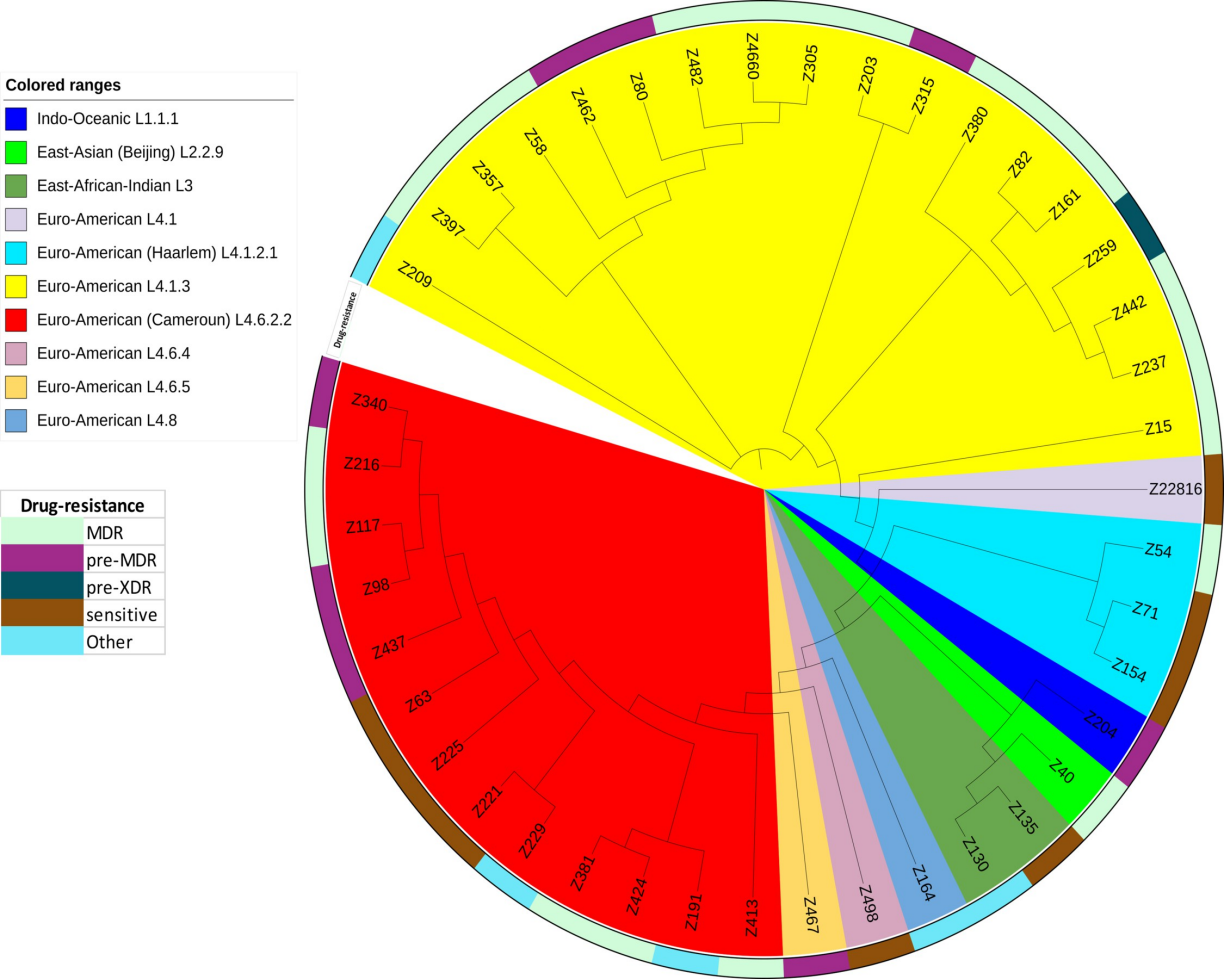
Core genome sequence-based phylogenetic tree displaying the sub-lineages of 42 *M*. *tuberculosis* isolates found in Niger. The colours of the isolates represent the resistance drugs of the sub-lineages. Phylogenetic trees based on the core genome were generated using FastTree 2.1.10_1 software (https://ngphylogeny.fr/tools/tool/271/form) using GTR model and bootstrap option with 1000 replicates. Tree was visualized using the iTOL online website.

During the two-year study period (13 isolates in 2016 and 29 isolates in 2017 in Niger), a total of 42 *M*. *tuberculosis* isolates were investigated in this study. Of the 42 isolates (41 pulmonary TB, one pleural fluid), 25 were MDR cases, 15 were new cases and two were relapsing cases (Figs 1 and 2). Sequencing reads indicated that they belonged to four *M*. *tuberculosis* lineages: Indo-Oceanic L1 (n = 1) (2.3%), East-Asian (n = 1) (2.3%), East-African Indian L3 (n = 2) (4.7%) and Euro-American L4 (n = 38) (90.4%) (S3 and S4 Tables); and 10 sub-lineages: The Indo-Oceanic lineage consisted of one sub-lineage L1.1.1 (n = 1) and the East Asian genotype L2 (Beijing) included one isolate in sub-lineage L2.2.9 (B0/W148) (2.3%). We detected two isolates belonging to the same East African Indian sub-lineage L3; while Euro-American L4 genomes were distributed in seven sub-lineages: the L4.1.3 sub-lineage comprised 18 isolates (47.3%), followed by the L4.6.2.2 (Cameroon) sub-lineage (n = 13 isolates) (34.2%) and the L4.1.2.1 sub-lineage (Haarlem) (n = 3 isolates) (7.8%). Finally, we identified one isolate in each of the following sub-lineages: L4.1 (2.6%), L4.6.4 (2.6%), L4.6.5 (2.6%) and L4.8 (2.6%) (Fig 2 and S3 and S4 Tables).

### Antibiotic susceptibility profile

Antibiotic profiling found 8/42 (19%) *in silico* susceptible *M*. *tuberculosis* isolates and 34/42 (81%) *M*. *tuberculosis* isolates exhibiting at least one predicted antibiotic resistance (Fig 2 and S2 Table). The predicted resistant strains comprised one pre-XDR isolate belonging to sub-lineage L4.1.3, twenty MDR isolates (20/34; 58.8%) belonging to sub-lineages L4.1.2.1 (n = 1/3), L4.1.3 (n = 13/18), L4.6.2.2 (n = 5/13) and L2.2.9 (n = 1/1), eight pre-MDR isolates (8/34; 23.5%) belonging to sub-lineages L4.1.3 (n = 3/18), L4.6.2.2 (n = 3/13), L4.6.5 (n = 1/1) and L1.1.1 (n = 1/1) and five (5/34; 14.7%) unclassified isolates exhibiting resistance to one or three antibiotics belonging to L3 (n = 1/2), L4.1.3 (n = 1/18), L4.6.2.2 (n = 2/13), L4.6.4 (n = 1/1) and L4.8 (n = 1/1) (S2 Table).

In detail, the predominant *M*. *tuberculosis* sub-lineages L4.1.3 was divided into 13 MDR isolates (13/18; 72.2%), three pre-MDR isolates (3/18; 16.6%), one pre-XDR (1/18; 5.5%) and one other drug resistant type isolate. Moreover, the *M*. *tuberculosis* sub-lineage L4.6.2.2 was divided into five MDR isolates (n = 5/13; 38.4%), three pre-MDR isolates (n = 3/13; 23%) and two other drug resistant type isolates.

Regarding bedaquiline-clofazimine susceptibility profile, we detected no mutation in the *atp*C, *atp*E and rv0678 genes. However, two L14R mutations (deleterious using PROVEAN with a score of -4.483) and D286G (neutral using PROVEAN with a score of -1.389) were detected in the rv1979c gene for *M*. *tuberculosis* Z204 (pre-MDR) belonging to sub-lineage L1.1.1. Other D283G mutations (deleterious using PROVEAN with a score of -2.935) were detected in the rv2535c gene for *M*. *tuberculosis* Z237 belonging to sub-lineage L4.1.3 (MDR). A specific amino acid insertion R473_V474insR (neutral using PROVEAN with a score of -0.092) was detected in the rv1979c gene, in common with *M*. *tuberculosis* sub-lineage L4.6.2.2 (S2 Table). These results showed that the *M*. *tuberculosis* sub-lineage L2.2.9 was detected as MDR.

Regarding ethionamide susceptibility profile, resistance mutation in *fab*G1 gene (c.-8T>C) was detected in Z58 (MDR), Z467 (pre-MDR), Z437 (pre-MDR) belonging respectively to sub-lineage 4.1.3 Euro-American, sub-lineage 4.6.5 Euro-American and sub-lineage 4.6.2.2 Cameroon. Other mutation (p.Arg463Ser) and deletion (c.878_878del) were detected in the *eth*A gene for *M*. *tuberculosis* Z380 (MDR) and Z164 (other) respectively belonging to sub-lineage 4.1.3 Euro-American and sub-lineage 4.8 mainly T. (S3 Table)

Regarding fluoroquinolone susceptibility profile, *gyr*B gene resistance mutations were detected in *M*. *tuberculosis* Z259 (pre-XDR, sub-lineage 4.1.3 Euro-American) and Z191 (other, sub-lineage 4.6.2.2 Cameroon) and one *gyr*A mutation in *M*. *tuberculosis* Z229 (other, sub-lineage 4.6.2.2 Cameroon). Regarding capreomycin, two resistance mutations were detected in *tly*A gene for *M*. *tuberculosis* Z467 (pre-MDR, sub-lineage 4.6.5 Euro-American) and *M*. *tuberculosis* Z204 (pre-MDR, sub-lineage 1.1.1 EAI). Finally, no resistance mutations detected for amikacin and kanamycin antibiotics.

## Discussion and conclusion

This first ever WGS-based analysis of clinical *M*. *tuberculosis* isolates in Niger provided an overview of the population structure and genetic distribution of *M*. *tuberculosis* complex isolates from pulmonary and pleural fluid patients and a TB susceptibility profile in Niger.

We identified four human-adapted lineages: Indo-Oceanic L1, East-Asian L2, East-African Indian L3, and Euro-American L4, which was largely predominant, as previously reported in Niger [37,38] and the bordering West African countries [39,40] including Benin [10], Burkina Faso [41], Chad [42], Mali [4,43] and Nigeria [44,45].

This study offered an unprecedented yet preliminary opportunity to map the geographical distribution of the 42 *M*. *tuberculosis* isolates in Niger (Fig 1). Only *M*. *tuberculosis* sub-lineage L.4.6.2.2 was observed in the regions of Zinder (two isolates) and Tillabéri (one isolate) and only *M*. *tuberculosis* sub-lineage L4.1.3 was observed in the region of Tahoua (five isolates) whereas we observed a greater distribution of the *M*. *tuberculosis* sub-lineage L2.2.9, L3, L4.1.3 and L4.6.2.2 in the regions of Maradi (11 isolates) and the *M*. *tuberculosis* sub-lineage L.1.1.1, L3, L4.1, L4.1.2.1, L4.1.3, L4.6.4, L4.6.5, L4.8 and L4.6.2.2 in the regions of Niamey (23 isolates). Data study showed that the sub-lineages L4.1.3 and L4.6.2.2 were predominant in Niger and were observed in all the five regions (Niamey, Maradi, Tahoua, Tillabéry and Zinder) (Figs 1 and 2 and S3 Table). These two predominant *M*. *tuberculosis* sub-lineages L4.1.3 and L4.6.2.2 were present in Niger as dangerous sub-lineages with XDR isolate (5.5%), MDR (72.2%), pre-MDR (16.6%) of sub-lineages L4.1.3 and with MDR isolates (38.4%), and pre-MDR (23%) of sub-lineage L4.6.2.2. Using the available database of Tb-profiler (https://tbdr.lshtm.ac.uk/sra) and the available genomes results in West Africa [46–50], we observed 99/8,139 (1.2%) isolates belonging to sub-lineage L4.1.3 (25/8,139; 0.3%) and L4.6.2.2 (74/8,139; 0.9%) in sub-Saharan Africa.

Regarding *M*. *tuberculosis* L4.1.3, we observed 25 *M*. *tuberculosis* sub-lineage L4.1.3 isolates distributed in Côte d’Ivoire (21/47 isolates; 44.6%), Nigeria (1/56 isolate; 1.8%) and Republic of the Congo (3/139 isolates; 2.15%). These isolates were divided into 21/25 MDR isolates (84%), 1/25 Pre-XDR isolate (4%), 1/25 pre-MDR isolate (4%) and 2/25 sensitive isolates (8%). The L4.1.3 had previously been detected in the West African countries. These data suggest that the MDR-TB sub-lineage L4.1.3 strain is also emerging in Niger (S5 Table). Regarding *M*. *tuberculosis* sub-lineage L4.6.2.2, we observed 74 isolates distributed in Ghana (15/458 isolates; 3.3%), Liberia (3/51; 5.8%), Malawi (3/2324; 0.12%), Democratic Republic of the Congo (5/329; 1.5%), Nigeria (29/56; 51.7%) and Côte d’Ivoire (16/47; 34%). These isolates were divided into 16/74 MDR isolates (21.6%), 6/74 other resistance isolates (8,1%), and 49/74 sensitive isolates (66.2%) (S6 Table).

The Indo-Oceanic lineage consisted of one sub-lineage L1.1.1 (n = 1; one other drug-resistant type isolate), and East-African Indian L3 (n = 2; 2 sensitive isolates) in this study, and it has been reported in Niger the low prevalence of these lineages [37].

The East Asian genotype L2 (Beijing) includes one isolate of the *M*. *tuberculosis* sub-lineage L2.2.9 (B0/W148) (2.3%). To our knowledge this data has been reported for the first time in this study and suggests that the Beijing L2.2.9 strains are also commonly described as an emerging genotype in West African countries such as Niger. Moreover, recent round-trip migrations to and from China may also have played an important role in emerging and rising frequency of Beijing lineage strains in Niger.

Regarding second-line drugs, this study showed that there is no resistance to amikacin and kanamycin, which allows its use in treatment. In addition, 95.2% of isolates were susceptible to the antibiotics clofazimine and bedaquiline. These data support the repurposing of anti-leprosy antibiotics as antituberculosis treatments [51]. More, the specific amino acid insertion detected was previously reported as specific insertion for sub-lineage L4.6.2.2 (Genotype Cameroon) [52]. Despite the small sample size, our study provides insight into the genomic diversity of lineages of *M*. *tuberculosis* and the drug-resistant epidemic in Niger. This study indicates the need to be aware of infection control procedures in healthcare establishments and within the population, and to carry out genomic epidemiological surveillance in Niger.

## Supporting information

S1 Table Mapping data of output sequencing reads using *M*. *tuberculosis* H37Rv (NC_000962.3) as the reference genome. (XLSX)

S2 Table Antibiotic susceptibility profile of the isolates and mapping approach against reference *Mycobacterium tuberculosis* H37Rv genes *in silico*. (XLSX)

S3 Table Lineages and sublineages identification of *Mycobacterium tuberculosis* isolates in Niger. (XLSX)

S4 Table Available sublineages data of *Mycobacterium tuberculosis* isolates in Niger (This study) and other Sub-Saharan Africa countries. (XLSX)

S5 Table Susceptibility profile of sublineage L4.1.3 isolates in Niger and other Sub-Saharan Africa countries. (XLSX)

S6 Table Susceptibility profile of sublineage L4.6.2.2 isolates in Niger and other Sub-Saharan Africa countries. (XLSX)
